# Sleep quality, anxiety, symptoms of depression, and caregiver burden among those caring for patients with Dravet syndrome: a prospective multicenter study in Germany

**DOI:** 10.1186/s13023-023-02697-3

**Published:** 2023-04-29

**Authors:** Margarita Maltseva, Susanne Schubert-Bast, Johann Philipp Zöllner, Thomas Bast, Thomas Mayer, Sarah von Spiczak, Susanne Ruf, Regina Trollmann, Markus Wolff, Frauke Hornemann, Kerstin A. Klotz, Julia Jacobs, Gerhard Kurlemann, Bernd A. Neubauer, Tilman Polster, Steffen Syrbe, Astrid Bertsche, Ulrich Bettendorf, Gerhard Kluger, Silke Flege, Felix Rosenow, Lara Kay, Adam Strzelczyk

**Affiliations:** 1 Epilepsy Center Frankfurt Rhine-Main, Department of Neurology, Goethe-University Frankfurt, Schleusenweg 2-16, 60528 Frankfurt am Main, Germany; 2 Center for Personalized Translational Epilepsy Research (CePTER), Goethe-University Frankfurt, Frankfurt am Main, Germany; 3 Department of Neuropediatrics, Goethe-University Frankfurt, Frankfurt am Main, Germany; 4 Epilepsy Center Kork, Kehl-Kork, Germany; 5 Faculty of Medicine, University of Freiburg, Freiburg im Breisgau, Germany; 6 Epilepsy Center Kleinwachau, Dresden-Radeberg, Germany; 7 Northern German Epilepsy Centre for Children and Adolescents, Kiel-Raisdorf, Germany; 8 Department of Neuropediatrics, University of Tübingen, Tübingen, Germany; 9 Department of Neuropediatrics, Friedrich-Alexander University, Erlangen, Germany; 10 Center of Pediatric Neurology, Vivantes Hospital Neukoelln, Berlin, Germany; 11 Swiss Epilepsy Center, Klinik Lengg AG, Zürich, Switzerland; 12 Department of Neuropediatrics, Leipzig University Hospital for Children and Adolescents, Leipzig, Germany; 13 Department of Neuropediatrics and Muscle Disorders, Medical Center – University of Freiburg, Freiburg im Breisgau, Germany; 14 Department of Pediatrics and Clinical Neurosciences, Cumming School of Medicine, University of Calgary, Calgary, AB Canada; 15 St. Bonifatius Hospital, Lingen, Germany; 16 Department of Neuropediatrics, Justus-Liebig-University Giessen, Giessen, Germany; 17 Epilepsy Center Bethel, Bielefeld, Germany; 18 Division of Pediatric Epileptology, Center for Pediatrics and Adolescent Medicine, University Hospital Heidelberg, Heidelberg, Germany; 19 Department of Neuropediatrics, University Hospital for Children and Adolescents, Rostock, Germany; 20 Department of Neuropediatrics, University Hospital for Children and Adolescents, Greifswald, Germany; 21 Neuropediatric Practice, Hirschaid, Germany; 22 Clinic for Neuropediatrics and Neurorehabilitation, Epilepsy Center for Children and Adolescents, Schoen Clinic Vogtareuth, Vogtareuth, Germany; 23 Research Institute “Rehabilitation, Transition, and Palliation”, PMU Salzburg, Salzburg, Austria; 24 Dravet Syndrom e.V., Markkleeberg, Germany

**Keywords:** Epilepsy, Seizure, Quality of life, Encephalopathy

## Abstract

**Background:**

This study measured sleep quality among caregivers of patients with Dravet syndrome (DS) and assessed the impacts of mental health problems and caregiver burden on sleep quality.

**Methods:**

This multicenter, cross-sectional study of patients with DS and their caregivers throughout Germany consisted of a questionnaire and a prospective 4-week diary querying disease characteristics, demographic data, living conditions, nocturnal supervision, and caregivers’ work situations. Sleep quality was assessed using the Pittsburgh Sleeping Quality Index (PSQI). The Hospital Anxiety and Depression Scale (HADS) and the Burden Scale for Family Caregivers (BSFC) were used to measure anxiety, symptoms of depression, and caregiver burden.

**Results:**

Our analysis included 108 questionnaires and 82 four-week diaries. Patients with DS were 49.1% male (n = 53), with a mean age of 13.5 ± 10.0 years. Caregivers were 92.6% (n = 100) female, with a mean age of 44.7 ± 10.6 years. The overall mean PSQI score was 8.7 ± 3.5, with 76.9% of participants (n = 83) scoring 6 or higher, indicating abnormal sleep quality. The HADS for anxiety and depression had overall mean scores of 9.3 ± 4.3 and 7.9 ± 3.7, respectively; 61.8% and 50.9% of participants scored above the cutoff value of 8 for anxiety and depression, respectively. Statistical analyses revealed caregiver anxiety levels and patients’ sleep disturbances as major factors influencing PSQI scores. The overall mean BSFC score of 41.7 ± 11.7 indicates a moderate burden, with 45.3% of caregivers scoring 42 or higher.

**Conclusions:**

Sleep quality is severely affected among caregivers of patients with DS, correlating with anxiety, comorbidities, and patients’ sleep disturbances. A holistic therapeutic approach should be implemented for patients with DS and their caregivers, focusing on the sleep quality and mental health of caregivers.

*Trial registration*: German Clinical Trials Register (DRKS), DRKS00016967. Registered 27 May 2019, http://www.drks.de/DRKS00016967

## Key points

1. Sleep quality is severely affected in caregivers of patients with Dravet syndrome (DS).
2. Anxiety level, caregiver burden, and patients’ comorbidities have major impacts on caregiver sleep quality.
3. We recommend a holistic therapy approach beyond seizures, including mental health challenges faced by caregivers of patients with DS.
4. The holistic therapeutic approach should target caregivers’ sleep disorders and any concomitant causes.

## Background

Dravet syndrome (DS) is a rare developmental and epileptic encephalopathy [1, 2]. Patients experience refractory epilepsy and numerous non-epileptic manifestations, such as impaired cognition, speech impairments, and delayed motor and behavioral development [3, 4]. The incidence of DS is estimated to be 1 in 15,000 live births, with a prevalence of 2 in 100,000 people [5–7]. In most patients, DS is caused by a mutation in the sodium channel protein type 1 subunit alpha (*SCN1A*) gene, which encodes a voltage-dependent sodium channel (Nav1.1) [8, 9].

Frequent, prolonged seizures develop during the first year of life in otherwise normal infants and are typically triggered by fever. Mortality in DS is high due to an increased risk of sudden unexpected death in epilepsy and frequent episodes of status epilepticus (SE) [10, 11].

The major focus of research in DS has been on the development and treatment of refractory epilepsy and associated clinical and pathophysiological issues. Recently, studies have begun to address the impacts of DS on caregivers and the family environment [12–14]. An international survey of 256 parents and caregivers identified impacts on siblings (74%) and possible depression in caregivers (66%) as major issues associated with caring for patients with DS, requiring more extensive research [15]. Another survey of 34 primary caregivers highlighted anxiety; depression; impacts on physical and emotional wellbeing; and time constraints as major problems encountered in caring for patients with DS [16]. Jensen et al. identified sleep deprivation, reduced mental health, deterioration of social relationships, and financial burden as major concerns among caregivers [17]; they authored a paper highlighting some of the open research questions in the field of DS, including the psychosocial impacts of caring for patients with DS [18]. A recent study by Nabbout et al. analyzed survey responses from 87 caregivers of patients with DS in France, highlighting the broad social and economic impacts on caregivers, especially mothers [19]. Although caregivers have reported sleep disturbances as critical factors impacting the quality of life of patients with DS who experience frequent seizure-related and non-seizure-related-awakenings, no comparable studies have examined the impacts of sleep disturbances on the quality of life of caregivers[20] or the various methods parents use to monitor their children’s sleep [21].

To address some of these identified open research questions, we conducted a study focusing on sleep quality among caregivers of DS patients. To the best of our knowledge, this study represents the first attempt to quantify sleep issues among caregivers of patients with DS using the well-established Pittsburgh Sleep Quality Index (PSQI), which will allow us to contextualize and evaluate the severity of previously identified sleep and mental health issues experienced by caregivers.

## Material & methods

This multicenter, cross-sectional study enrolled primary caregivers of patients with DS throughout Germany (via specialists in Berlin, Bielefeld, Dresden, Erlangen, Frankfurt, Freiburg, Giessen, Heidelberg, Hirschaid, Kiel, Kork, Leipzig, Lingen, Münster, Rostock, Tübingen, Vogtareuth and through the German DS patient advocacy group [Dravet Syndrom e.V., Markkleeberg, Germany]). Written informed consent was obtained from the parents or legal guardians of patients with DS. The study obtained ethics approval from Goethe-University Frankfurt, Frankfurt am Main, and was registered with the German Clinical Trials Register (DRKS00016967). The Strengthening and Reporting of Observational Studies in Epidemiology (STROBE) guidelines were closely followed during the conduct and reporting of this study [22].

A combined survey, consisting of a previously validated retrospective questionnaire and a prospective diary [23], was administered to enrolled participants in Germany in 2019. Both the questionnaire and diary were linked to an anonymous identification number. The questionnaire consisted of 57 questions relating to disease characteristics (seizures, medical treatment, comorbidities, care level, disability degree), demographic data, the living conditions of both patients and caregivers, the work situation of caregivers, and nocturnal supervision details (monitoring and use of devices). This data was supplemented with information on the presence or absence of a disability (Grad der Behinderung) and the child’s care level (Pflegegrad), both provided by the caregiver but having been independently assessed by the social security office (Versorgungsamt) and the nursing care insurance system (Pflegeversicherung) that includes a mandatory medical assessment by a physician [13]. The questionnaire included both closed and open-ended questions; closed questions included responses that were list-based, multiple-choice options, or Likert scales.

In addition, the PSQI, Hospital Anxiety and Depression Scale (HADS), and Burden Scale for Family Caregivers (BSFC) were included in the questionnaire. The PSQI is a widely used, well-validated measure of sleep that consists of seven subcategories: sleep quality, duration, latency, efficiency, disturbances, daytime dysfunction, and use of sleep medication. Scores (range: 0–21) above 5 are considered to indicate a sleep problem [24, 25].

The HADS, a validated and well-known tool for measuring anxiety and depression consisting of 14 items, was used to assess mental health. Higher scores on the subscales for anxiety (HADS-A, range: 0–21) and depression (HADS-D, range: 0–21) indicate higher levels of stress. In this study, we used cutoff values of 8 for both subscales to indicate possibly abnormal levels of depression and anxiety. Values of 11 or above were considered indicative of a high likelihood of depression or anxiety. According to various studies in the literature, a cutoff value of 8 represents the optimal balance between sensitivity and specificity for the HADS [26, 27].

The BSFC is a 28-item instrument used to quantify the perceived burden of family caregivers. The total score (range: 0–84) indicates three levels of burden: a total score ≤ 41 correlates with no to mild stress with no increased risk of developing psychosomatic symptoms; a score of 42–55 indicates moderate stress, associated with an increased risk of developing psychosomatic symptoms; and a score ≥ 56 indicates severe to very severe stress, associated with a very high risk of developing psychosomatic symptoms [28, 29]. The questionnaire had to be filled by the main caregiver who is defined as a the person spending the most time with caring for the patient with Dravet syndrome. The main caregiver had to complete the questionnaire, answering questions about a retrospective time period of three months. The estimated time for completion was about one hour.

The prospective diary collected data on seizures, monitoring, and emergency treatment for one month and was used to validate diurnal and nocturnal seizure frequency data reported in the retrospective questionnaire.

Statistical analysis was conducted using IBM SPSS version 27 (IBM Corp., Armonk, NY, USA). Variables of interest were summarized using the mean, median, range, and standard deviation (SD). Pearson’s correlation coefficient was calculated for continuous variables. Correlations with coefficients < 0.3 were not considered for interpretation based on Cohen’s definitions (< 0.3 is a low correlation; 0.3–0.5 is a medium correlation; > 0.5 is a high correlation) [30]. The Wilcoxon signed-rank test was performed to compare factors influencing sleep quality, anxiety, depression, and caregiver burden. A two-sided significance level of *p* = 0.05 was used in all statistical analyses. A Chi-square analysis was performed to compare our cohort with the normal population.

## Results

### Patient and caregiver characteristics

In total, 108 questionnaires and 82 prospective diaries collected from patients and their caregivers were included in our analyses. Patients with DS had a mean age of 13.5 years (SD 10.0 years, median 10.8 years), and 53 (49.1%) were male. The mean age of mothers was 44.7 years (SD 10.6 years, median 42 years), and the mean age of fathers was 47.3 years (SD 10.6 years, median 53.5 years). Most of the primary caregivers (92.6%, n = 100) were women, whereas 7.4% (n = 8) were men.

#### Clinical characteristics

A pathogenic *SCN1A* variant was reported in almost all patients (n = 104; 96.3%, in 4 the results of genetic testing were not available). Caregivers reported a mean of 8.5 seizure days per month (SD 9.8 seizure days; median 4 seizure days; range 0–30 seizure days; average based on reports for the previous 3 months), which was comparable to the prospectively recorded mean of 8.6 seizure days (SD 10.8 seizure days; median 3 seizure days; range 0–30 seizure days; n = 82). Nocturnal seizures were reported for 71.4% (n = 77) of patients in the questionnaire and for 52.4% (43/82) of patients in the diary, with a mean of 5.6 nocturnal seizure days per month (SD 9.3 nocturnal seizure days; median 1 nocturnal seizure day; range 0–30 nocturnal seizure days; n = 82) among patients who submitted diaries. The mean frequency of nocturnal seizures was 8.9 (SD 25.6; median 1; range 0–208) seizures per month. Details regarding overall generalized tonic–clonic seizure (GTCS) and nocturnal seizure frequencies are presented in Table 1.

**Table 1:** Sociodemographic and clinical characteristics of patients and caregivers

	All patients n = 108
Patient characteristics	
Age in years^1^	13.5 ± 10.0, range 1.2–46.2
Sex	% (n)
Male	49.1 (53)
Female	50.9 (55)
Genetics	% (n)
* SCN1A*-mutation	96.3 (104)
Unknown or not available	2.8 (3)
Epilepsy characteristics	
Seizure days per month^1^	8.5 ± 9.8 range 0–30
Nocturnal seizures^1^	8.4 ± 25.6 range 0–208
Nocturnal seizure frequency	% (n)
At least one per night	9.3 (10)
At least one per week	21.3 (23)
At least one per month	16.7 (18)
At least one per 6 months	11.1 (12)
At least one per year	13.0 (14)
No seizures for more than a year	25.9 (28)
GTCS frequency	% (n)
At least one per day	4.6 (5)
At least one per week	26.9 (29)
At least one per month	25.0 (27)
At least one per 6 months	17.6 (19)
At least one per year	11.1 (12)
No seizures for more than a year	13.0 (14)
Mean number of ASMs^1^	3.2 ± 1.2 range 1–5
Living conditions	% (n)
With mother and father	71.3 (77)
Only with mother	12.0 (13)
In a nursing home	2.8 (3)
Other	13.9 (15)
Caregiver characteristics	
Age of mother (years, n = 108)^1^	44.7 ± 10.6
Age of father (years, n = 108)^1^	47.3 ± 10.6
Mother's professional situation	% (n)
Housemaker	28.7 (31)
Employed	46.3 (50)
Student	1.9 (2)
Receiving pension	8.3 (9)
Father's professional situation	% (n)
Housemaker	0.9 (1)
Employed	85.2 (92)
Student	0.0 (0)
Receiving pension	5.5 (6)
Change in mother's professional situation due to child's DS	% (n)
No change	23.1 (25)
Yes, quit her work	33.3 (36)
Yes, different job	10.2 (11)
Yes, reduction of working hours	26.9 (29)
Change in father's professional situation due to child's DS	% (n)
No change	88 (95)
Yes, quit his work	0.9 (1)
Yes, different job	1.9 (2)
Yes, reduction of working hours	2.8 (3)
Use of pediatric intensive care service	% (n)
Yes	16.7 (18)
No	81.5 (88)

^1^Mean ± standard deviation; ASM, anti-seizure medication; DS, Dravet syndrome

Patients were treated with a mean of 3.2 anti-seizure medications (ASMs; SD 1.2; median 3; range 1–5). Valproate (n = 68; 63.0%), clobazam (n = 59; 54.6%), bromides (n = 47; 43.5%), stiripentol (n = 41; 38.0%), and topiramate (n = 28; 25.9%) were the most frequently used ASMs.

**Fig. 1:**
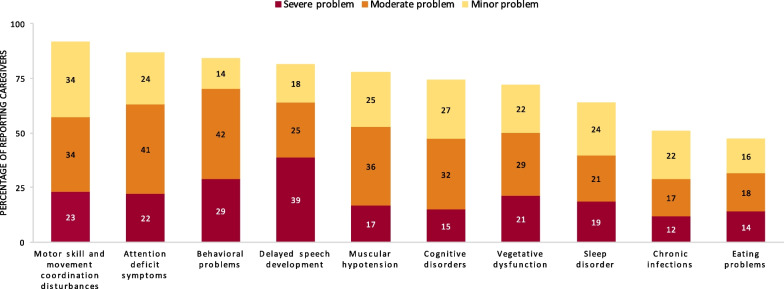
Patient comorbidities and their impacts as reported by caregivers in the past 3 months (source: questionnaire, n = 108)

Patients presented with a range of comorbidities, the impacts of which were ranked from “minor” to “severe” by caregivers (Fig. 1). Motor skills and movement coordination disturbances (n = 92, 85.2%), attention-deficit symptoms (n = 87, 80.6%) and behavioral problems (n = 84, 77.8%) were reported by most caregivers. Most impairments were rated by at least half of caregivers as being moderate or severe, including behavioral problems (70.5%), delayed speech development (63.9%), attention-deficit symptoms (62.9%), motor skill and movement coordination disturbances (57.4%), muscular hypotension (52.8%), and vegetative dysfunction (50.0%). Sleep disorders in patients with DS were reported by 59.3% (n = 64) of caregivers and were rated “moderate” or “severe” by 21.3% and 18.5% of caregivers, respectively. Level I–V care was required by 94% (n = 101) of patients using the Pflegebedürftigkeit scale (German “need for care”). Although the level-I–V criteria were not met by 2% of patients, the caregivers of these patients indicated that these patients were in need of care. Only 4.6% of patients were not categorized as in need of care, and 95.4% percent (n = 102) had a severely disabled pass.

#### Living conditions and nocturnal supervision

Overall, 71.3% of patients lived in one household with both parents, and 12.0% lived only with their mothers. Most fathers (85.2%) worked, whereas employment was only reported by 46.3% of mothers. Most mothers (70.4%) reported a change in their work situation to accommodate their child with DS, whereas work changes were reported by six fathers (5.6%) (Table 1). One-third of patients with DS (n = 36; 33.3%) slept in their parents’ bedrooms, including 24 who slept in the same bed as their primary caregiver. In 16.7% (n = 18) of cases, the caregivers were supported by a pediatric intensive care service. Monitoring devices were used regularly by 75.9% (n = 82) of caregivers, and among this group, 87.8% (n = 72/82) reported daily monitoring. Pulse oximeters (n = 53/82; 64.6%), baby monitors (n = 53/82; 64.6%, 38 of these include camera functions), thermometers (n = 28/82; 24.1%), and Epi-Care® (n = 22/82; 26.8%) were the most frequently used monitoring devices.

### Pittsburgh sleep quality index (PSQI), hospital anxiety and depression scale (HADS), and burden scale for family caregivers (BSFC)

PSQI scores of 6 or above were reported by 76.9% (n = 83) of primary caregivers, indicative of abnormal sleep quality. In 30.6% (n = 33) of caregivers, a score of 11 or above was achieved, indicating severe sleep disorders. The overall mean PSQI score of caregivers was 8.7 (SD 3.5; median 8.5; range 2–16), compared with a mean score of 5 (SD 3.4) among the general German population. Analysis of subcategories was feasible in 106 to 108 caregivers, and details for caregivers and comparisons with the general German population are provided in Table 2. Caregivers scored worse in all subcategories than the general population except for the subcategory “use of sleep medication.” Caregivers spent an average of 7.5 h (SD 0.9 h; median 7.5 h) in bed but slept for only 5.8 h (SD 1.1 h; median 6 h). A sleep duration of at least 6 h was reported by 66.7% of caregivers, which is significantly lower than the 85.8% of the general population who sleep for at least 6 h (*p* < 0.001).

**Table 2:** Pittsburgh sleep quality index (PSQI), hospital anxiety and depression scale (HADS), and burden scale for family caregivers (BSFC)

	Main caregivers of patients with Dravet syndrome						General German population		
	n	Mean	SD^3^	Minimum	Median	Maximum	n	Mean	SD^3^
PSQI^3^	108	8.7	3.5	2	8.5	16	9284	5	3.4
Subjective sleep quality	108	1.7	0.6	0	2	3		1.1	0.7
Sleep latency	108	1.3	1.1	0	1	3		1.1	0.9
Sleep duration	107	1.6	1.2	0	1	3		0.6	0.8
Sleep efficiency	106	1.2	1.1	0	1	3		0.7	1.0
Sleep disturbance	108	1.3	0.5	0	1	2		0.6	0.6
Use of sleep medication	107	0.1	0.4	0	0	3		0.1	0.6
Daytime dysfunction	108	1.6	0.8	0	2	3		0.8	0.7
Bedtime	106				22:00				
Time to sleep (min)	106	25.9	26.5		20				
Waking time	107				06:00				
Time spend in bed (h)	106	7.5	0.9		7.5				
Effective sleep time (h)	107	5.8	1.1		6				
Gender of primary caregiver 92.6% (n = 100) female, 7.4% (n = 8) male, mean age 45 years							56.6% female (n = 2481), male 43,7% (n = 1929)*		
HADS-A	108	9.3	4.3	1	9	20	4410	4.4^1^/5.0^2^	3.3^1^/3.6^2^
HADS-D	108	7.9	3.7	1	7	20	4410	4.8^1^/4.7^2^	4.0^1^/3.9^2^
BSFC	108	41.7	11.7	17	40	64	^1^male		
Pearson-Correlation of PSQI with		r	*p*-value				^2^female		
HADS-A	106	0.46	< 0.001				* mean age 50.3 years		
HADS-D	106	0.21	0.03						
BSFC	106	0.36	< 0.001						
PSQI	% (n)								
0–5	23.1 (25)								
6–10	46.3 (50)								
11–21	30.6 (33)								
HADS-A									
Normal (0–7)	38.2 (39)								
Borderline (8–10)	26.5 (27)								
Abnormal (11–21)	35.3 (36)								
HADS-D									
Normal (0–7)	49.1 (53)								
Borderline (8–10)	27.8 (30)								
Abnormal (11–21)	23.1 (25)								
BSFC									
None to mild (0–41)	54.6 (59)								
Moderate (42–55)	33.3 (36)								
Severe to very severe (56–84)	12.0 (13)								

^3^SD, Standart deviation; r, correlation-coefficient; PSQI, Pittsburgh Sleep Quality Index; HADS, Hospital Anxiety and Depression Scale

BSFC, Burden Scale for Family Caregivers

The HADS score for anxiety was normal (0–7) for 38.2% of caregivers (n = 39), borderline (8–10) for 26.5% of caregivers (n = 27), and abnormal for 35.3% of caregivers (n = 36), indicating a tendency toward severe anxiety symptoms. The overall mean HADS anxiety score was 9.3 (SD 4.3; median 9; range 1–20), compared with mean HADS anxiety scores of 4.4 (SD 3.3 for men) and 5.02 (SD 3.6 for women) among the general German population. The HADS score for depression was normal (0–7) for 49.1% of caregivers (n = 53), borderline (8–10) for 27.8% of caregivers (n = 30), and abnormal for 23.1% of caregivers (n = 25), indicating a tendency toward severe depression symptoms. The overall mean HADS depression score was 7.9 (SD 3.7; median 7; range 1–20), compared with mean HADS depression scores of 4.8 (SD 4.0 for men) and 4.7 (SD 3.9 for women) among the general German population [31]. In our study, 61.8% and 50.9% of caregivers reported scores above 8 for anxiety and depression, respectively, compared with the general German population, in which 21% and 23% scored above 8 on the anxiety and depression subscales, respectively.

The mean BSFC score was 41.7 (SD 11.7; median 40) and ranged from 17 to 64, indicating a moderate overall care burden. The BSFC indicated a mild burden (0–41) for 54.6% of caregivers (n = 59), a moderate burden (42–55) for 33.3% of caregivers (n = 36), and a severe or very severe burden (56–84) for 12.0% (n = 13) of caregivers.

### Correlations among factors influencing sleep quality, anxiety, depression, and caregiver burden

**Fig. 2:**
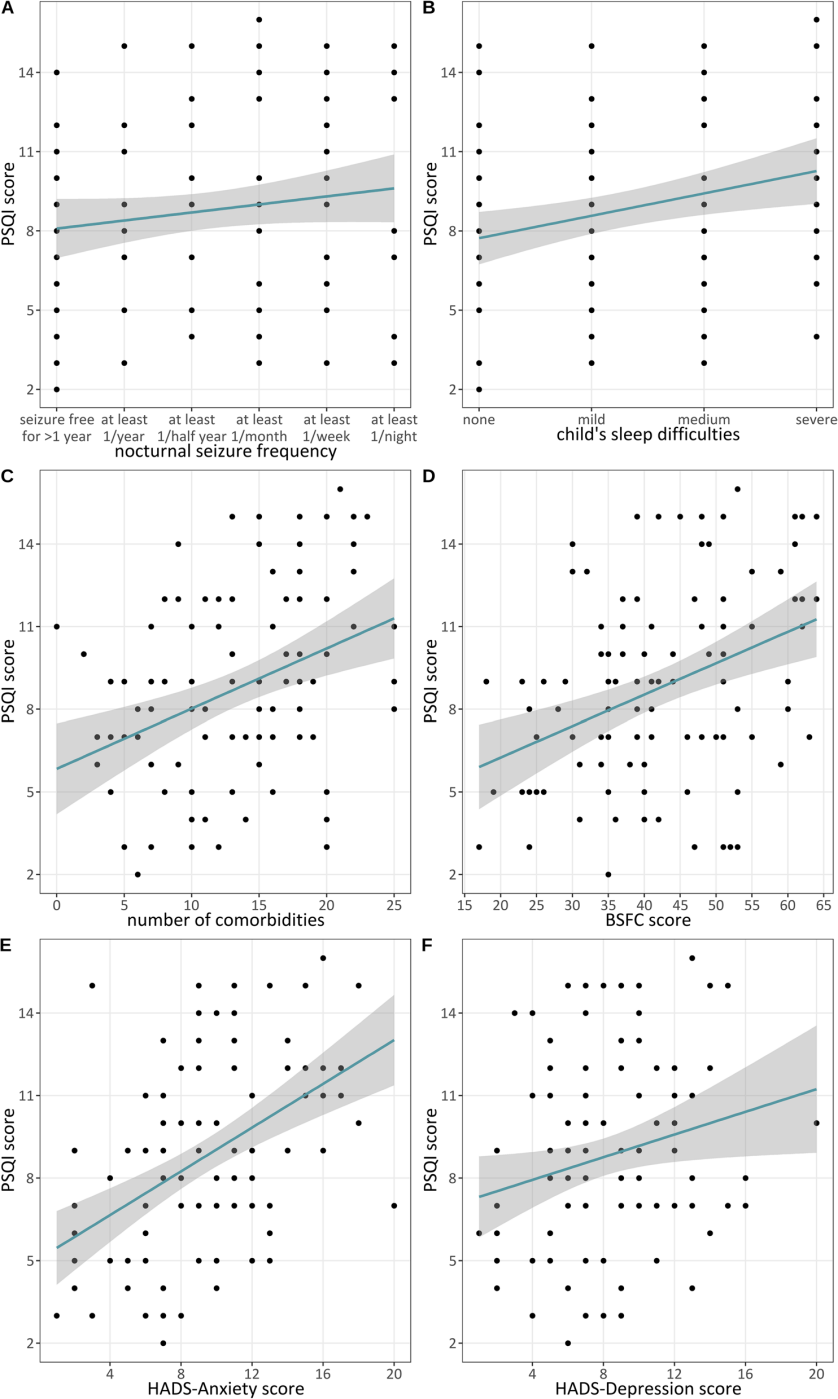
Impacts on the Pittsburgh Sleep Quality Index (PSQI), shown in regression variable graphs, associated with nocturnal seizure frequency (**A**), child’s sleep difficulties (**B**), the number of comorbidities (**C**), the Burden Scale for Family Caregivers (BSFC) score (**D**),and the Hospital Anxiety and Depression Scale (HADS) anxiety (**E**) and depression (**F**) scores

Univariate analyses of the impacts of clinical categories for patients with DS on the PSQI, HADS-A, HADS-D, and BSFC scores of caregivers are presented in Table 3. Higher PSQI scores were observed among caregivers who reported at least moderate sleep disturbances (*p* = 0.02) for patients with DS; correlations of PSQI scores with seizure frequency and other clinical characteristics or caregiver’s work situation did not achieve significance. The graphical representation of correlations between PSQI scores among caregivers and the nocturnal seizure frequency and sleep disturbances reported for patients with DS are presented in Fig. 2A and B, respectively. Higher HADS-A scores were observed among caregivers who reported at least moderate sleep disturbances (*p* = 0.03) and among caregivers who slept in the same bedroom as patients with DS (*p* = 0.03). Higher HADS-D scores were correlated with a higher frequency of GTCS incidents (*p* = 0.01) and were observed among caregivers who slept in the same bedroom as patients with DS (*p* = 0.01). Higher BSFC scores were observed for higher frequency of nocturnal seizures (*p* = 0.03), a higher frequency of GTCSs (*p* = 0.02), and moderate sleep disturbances (*p* = 0.02). Higher PSQI scores correlated significantly with a higher number of comorbidities (r = 0.367; *p* < 0.001) and higher BSFC (r = 0.358; *p* < 0.001), HADS-A (r = 0.456; *p* < 0.001), and HADS-D scores (r = 0.215; *p* = 0.026) (Fig. 2C–F).

**Table 3:** Univariate correlations among factors influencing sleep quality, anxiety, depression, and caregiver burden

	PSQI					HADS-Anxiety				HADS-Depression				BSFC			
	n	Mean	SD^1^	Median	*p*-value^2^	Mean	SD^1^	Median	*p*-value^2^	Mean	SD^1^	Median	*p*-value^2^	Mean	SD^1^	Median	*p*-value^2^
Number of ASMs																	
1–2 ASMs	28	8.3	3.1	9	0.57	9.4	4.8	9	0.88	7.1	3.6	7	0.22	40.3	12.8	39.5	0.54
≥ 3 ASMs	80	8.8	3.7	8		9.2	4.1	9		8.2	3.9	8		42.1	11.4	40.5	
Sex																	
Male	53	8.5	3.5	9	0.56	9.1	4.5	9	0.68	7.7	3.6	7	0.78	42.3	12.3	41	0.53
Female	55	8.9	3.6	8		9.5	4.1	9		8.1	4.1	7		41.1	11.2	40	
Frequency of nocturnal seizures																	
Daily or weekly	33	9.2	3.6	9	0.30	9.1	4.3	9	0.77	8.9	4.7	9	0.14	45.2	12.2	48	**0.03**
Once per month or less	75	8.4	3.5	8		9.4	4.3	9		7.4	3.2	7		40.1	11.2	39	
Frequency of GTCSs																	
Daily or weekly	34	9.1	3.5	9	0.39	9.9	3.9	10	0.24	9.3	3.9	9	**0.01**	45.4	11.9	45.5	**0.02**
Once per month or less	74	8.5	3.6	8		9.0	4.5	9		7.3	3.5	7		40.0	11.3	39	
Disabled person pass degree																	
< 80% degree of disability	14	8.6	2.4	9	0.91	9.2	4.8	8.5	0.70	6.8	3.4	7	0.25	37.4	12.5	34	0.10
≥ 80% degree of disability	94	8.7	3.7	8		9.3	4.2	9		8.1	3.8	8		42.3	11.5	41	
Need for care																	
Care level 1–3	51	8.7	3.3	9	0.75	9.8	4.4	9	0.34	8.3	3.3	8	0.19	42.0	12.4	40	0.87
Care level 4–5	57	8.6	3.7	8		8.8	4.1	9		7.6	4.1	7		41.4	11.1	41	
Help of pediatric intensive care service																	
No use of pediatric intensive care service	88	8.7	3.6	8.5	0.89	9.1	4.4	9	0.16	7.7	3.7	7	0.07	41.4	11.9	40	0.54
Use of pediatric intensive care service	18	8.5	3.3	8.5		10.5	3.7	10.5		9.4	3.4	9		43.4	11.6	43.5	
Sleep disturbance of the patients																	
No or minor sleep disturbances	65	7.8	3.2	8	**0.02**	8.5	4.1	8	**0.03**	7.5	3.8	7	0.08	39.5	11.4	39	**0.02**
Sleep disturbance at least moderate problem	43	10.0	3.5	10		10.4	4.4	10		8.6	3.7	9		44.9	11.5	45	
Use of monitoring devices																	
Yes	82	8.9	3.6	9	0.11	9.6	4.3	9	0.20	8.0	3.9	7	0.98	41.8	11.2	40	0.98
No	26	7.7	4.0	7		8.2	4.2	8.5		7.7	3.4	8		41.2	13.3	41	
Sleeping conditions																	
Patient sleeps in his/her own room	70	8.5	3.6	9	0.40	8.7	4.0	9	**0.03**	7.3	3.6	7	**0.01**	41.1	12.0	40	0.38
Patient sleeps in parents bedroom	36	9.2	3.6	8		10.7	4.6	10.5		9.4	4.2	9		43.6	10.7	42.5	
Patient has his/her own bed	12	9.4	4.1	8		9.5	5.7	9.5		7.6	4.2	7		39.4	11.7	35	
Patient sleeps in the conjugal bed	24	9.1	3.0	8.5		11.3	4.0	11		10.3	4.0	10.5		45.8	9.8	47.5	
Main cageriver's work situation																	
Yes	52	9.0	3.6	8.5	0.47	8.9	4.2	8.5	0.40	7.3	3.5	7.0	0.15	41.3	11.8	39.0	0.52
No	56	8.4	3.5	8.5		9.6	4.4	9.5		8.5	3.9	8.0		42.0	11.7	41	

Bold values highlight statistically significant results (*p* < 0.05)

^1^SD, standard deviation; ASM, anti-seizure medication; GTCS, generalized tonic–clonic seizure; PSQI, Pittsburgh Sleep Quality Index; HADS, Hospital Anxiety and Depression Scale; BSFC, Burden Scale for Family Caregivers

^2^of the Wilcoxon-W-Test

## Discussion

The majority of primary caregivers for patients with DS reported low sleep quality, which correlated with anxiety and depression symptoms and a high caregiver burden. Our findings support the notion that DS is a multifactorial disease presenting not only with refractory epilepsy but also with numerous symptoms and comorbidities that affect caregivers and the family environment [3]. Supportive therapy and services should be offered to a substantial proportion of caregivers to help them in their daily lives.

Our findings are in line with other studies reporting significant impacts experienced by the caregivers of patients with DS. Almost half of the caregivers (45.3%) in our cohort perceived at least a moderate burden as measured by the BSFC. In addition to high seizure and SE frequencies, numerous comorbidities affect behavior, motor skills, development, vegetative dysfunction, cognition, communication, and sleep in patients, which impact the quality of life for both patients and their caregivers [4, 15, 19, 32–34].

The totality of these impairments results in an overall disability level that affects the sleep quality of caregivers. As observed in our results, caregivers’ sleep quality is highly dependent on their anxiety level (r = 0.456; *p* < 0.001) and the overall impact of comorbidities (r = 0.367; *p* < 0.001), particularly sleep disturbances among patients with DS. Contrary to expectations, nocturnal seizures and monitoring had no significant impacts on the PSQI. However, a higher frequency of seizures resulted in a higher caregiver burden and a higher anxiety level.

Sleep disturbances among patients with DS have previously been addressed in the literature. Losito et al. retrospectively analyzed clinical and electroencephalogram data of patients with DS to detect sleep-related and nocturnal seizure clusters. They stated that the sleep quality for both the patient and their caregiver might be worsened by certain sleep patterns and nocturnal seizures occurring between the ages of 4 and 11 years, which are often underdiagnosed [35]. Licheni et al. demonstrated that sleep disturbances represent an important problem among patients with DS patients based on the outcomes of the Sleep Disturbance Scale for Children and a seizure questionnaire, although the incidence of sleep disturbances reported in their cohort was higher (75%) than that in ours (63.9%). Furthermore, they reported that most difficulties were associated with initiating and maintaining sleep, particularly among patients older than 20 years. Our study confirmed that sleep disturbances among patients with DS were an important risk factor for sleep deprivation and poor sleep quality among caregivers and elaborated on their impacts on anxiety levels and the perception of caregiver burden.

To our knowledge, this is the first study to use the PSQI to quantify sleep problems among caregivers of patients with DS. The PSQI is recommended in the current guidelines of the German Society for Sleep Medicine and Sleep Research[36] and has been shown to be reliable [37]. The proportion of poor sleepers in our cohort (76.9%) was double the proportion among the general German population [31]. Lower sleep quality in caregivers of patients with DS was positively correlated with anxiety symptoms and perceived caregiver burden; a minor, positive correlation was also observed with depressive symptoms. Moreover, Pearson’s coefficients indicated the highest correlations between sleep quality and anxiety level.

A study performed in Spain examined PSQI values among informal caregivers of individuals whose degree of dependence ranged from moderate to total due to various physical disabilities and mental disorders [38]. The study divided 201 caregivers into two groups according to their perceived burden (measured by the Caregiver Burden Inventory) and reported mean PSQI scores of 7.0 and 10.4 for those with low and high perceived burdens, respectively, whereas a control group had a mean PSQI score of 5.9. The PSQI values of caregivers for dependent individuals in Spain were similar to the results observed in our study. A high rate of sleep difficulties in young children with epilepsy and their parents was reported by Reilly et al. [39]; 62% of mothers and 44% of fathers reported poor sleep quality on the PSQI. However, the authors did not observe a significant difference between mothers of children with epilepsy and in a non-epilepsy-related neurodisability group [39]. There is a need to develop effective interventions for this population, taking into consideration of the role of child behavioral problems and parental mental health difficulties. A recent publication evaluated anxiety, depression, and sleep quality among parents of children with epilepsy in Southern China [40]. They reported a mean PSQI score of 6.9, which was significantly higher than the mean of 5.0 scored by the parents of healthy children. Another study examining caregivers of physically disabled children in Japan reported a mean total PSQI score of 4.9, with 34% of caregivers rated as poor sleepers [41]. Chu and Richdale examined the caregivers of children with developmental disabilities and found a mean PSQI score of 8.8 [42]. Our findings indicate that sleep quality is highly affected among caregivers of patients with DS, and compared with the caregivers of physically disabled children and of children with epilepsy, in general, the proportion of poor sleepers was larger among caregivers of patients with DS.

Villas et al. designed an online survey for the parents and caregivers of patients with DS to identify the top concerns among caregivers and establish approximate frequencies of characteristics and comorbidities associated with DS beyond seizures. They stated that an alarmingly high percentage of caregivers (80%) slept in the same bed as patients with DS for safety reasons, which could lead to significant sleep disturbances experienced by both the caregiver and the patient. Another parent-driven online survey, conducted by Van Nuland et al., queried patients' sleep disturbances and showed that 59% of parents used co-sleeping as a monitoring method. Although fewer caregivers in our cohort (33.3%) slept in the same bedroom or even the same bed as the patient (22.6%), a significant impact on depression and anxiety symptoms was observed among these individuals; however, these impacts were not directly apparent on the PSQI. Our cohort was similar to the cohort studied by Villas et al. (92% *SCN1A* mutations, median age 7–10 years, patients’ recruitment by means of an advocacy group) [9]; however, the surveys were conducted several years apart, and modern supervision devices might have replaced co-sleeping among our cohort.

Sleep quality should receive more attention in daily treatment, as the sleep quality among caregivers of patients with DS appears to be deeply affected, exceeding the effects reported for caregivers of patients with other rare diseases [43]. One possible approach is to examine the effects of brief behavioral sleep interventions. For example, one study examined interventions that included stimulus control, relaxation, cognitive therapy, and sleep hygiene elements, measuring self-reported sleep quality, depressive symptoms, and quality of life among 30 caregivers of patients with cancer, which showed that participants who received interventions improved PSQI scores compared with a control group [44]. Behavioral interventions have also been shown to be effective in primary insomnia [37]. The first intervention for improving sleeping problems is to inform caregivers about sleep hygiene tools that can easily be implemented in daily treatment routines. In addition, patients' sleep problems and the shared sleep situation between patients and caregivers should be taken into consideration. A more detailed analysis of nocturnal monitoring devices could provide a new study base for the presentation of alternatives to co-sleeping. Furthermore the introduction of new ASMs like cannabidiol and fenfluramine might have an effect on patients’ sleep quality [45–47], but this needs to be evaluated in future studies. Another approach to addressing sleep problems would be to target caregivers’ anxiety levels as the primary factor influencing PSQI scores.

The limitations of this study include the study design, as surveys are susceptible to response and recall biases. To limit recall biases, data from the questionnaire were validated using the prospective diary. Individual clinical examinations would have provided further insights into the precise contributions of depression or anxiety to quality of life, but these were beyond the scope of this questionnaire-based study. We have to emphasize that no clinical diagnosis of depression is made in caregivers based on our survey data. Therefore, only symptoms of depression and anxiety are discussed. Furthermore, the questionnaire was completed only by the main caregiver. We have considered interviewing both parents and even the siblings; however, the estimated time to answer the questionnaire was about one hour and interviewing both parents and even the siblings would exceed a reasonable amount of time spent on the study from the caregiver’s perspective. Potential selection bias must be considered due to differences in the willingness to participate in studies across different population strata. Moreover, regional or national particularities may have influenced the results; however, the use of established questionnaires and compliance with the STROBE criteria were applied to minimize these aspects to the extent that was reasonably possible [22].

Some categories, such as degree of developmental delay, were not defined and relied on caregivers' subjective assessments, which may not be representative of a physician's assessment. Furthermore, although the sample consisted of individuals recruited from a variety of sources (multiple clinics and centers across Germany and through the patient advocacy group), the sample may not have been representative of DS patients and their caregivers in Germany in general. One strength of this study was its sample size of 108 patients and caregivers, given the rarity of DS. However, to identify more predictors for sleep quality and mental status, larger sample sizes are needed.

## Conclusion

To our knowledge, this is the first study to quantify sleep quality among caregivers of patients with DS using the validated and well-established PSQI and to attempt to quantitatively address the underlying psychological issues associated with poor sleep quality. We have shown a decrease in sleep quality and increased levels of anxiety and depression among caregivers of patients with DS relative to the general population. The patient’s overall disability level, sleep disturbances, and caregiver’s anxiety level are major factors influencing sleep quality. We recommend implementing a therapeutic approach for patients with DS that goes beyond seizures and includes caregivers, focusing on sleep quality and the impacts of sleep quality on caregivers' mental health.

## Data Availability

The datasets analysed during the current study are available from the corresponding author on reasonable request.

## References

[1] Dravet C (2011). The core Dravet syndrome phenotype. Epilepsia.

[2] Strzelczyk A, Schubert-Bast S (2020). Therapeutic advances in Dravet syndrome: a targeted literature review. Expert Rev Neurother.

[3] Gataullina S, Dulac O (2017). From genotype to phenotype in Dravet disease. Seizure.

[4] Lagae L, Brambilla I, Mingorance A, Gibson E, Battersby A (2018). Quality of life and comorbidities associated with Dravet syndrome severity: a multinational cohort survey. Dev Med Child Neurol.

[5] Brunklaus A, Ellis R, Reavey E, Forbes GH, Zuberi SM (2012). Prognostic, clinical and demographic features in SCN1A mutation-positive Dravet syndrome. Brain.

[6] Rosander C, Hallbook T (2015). Dravet syndrome in Sweden: a population-based study. Dev Med Child Neurol.

[7] Wu YW, Sullivan J, McDaniel SS, Meisler MH, Walsh EM, Li SX, Kuzniewicz MW (2015). Incidence of Dravet syndrome in a US population. Pediatrics.

[8] Lee J, Lee C, Park WY, Lee J (2020). Genetic diagnosis of Dravet syndrome using next generation sequencing-based epilepsy gene panel testing. Ann Clin Lab Sci.

[9] Depienne C, Trouillard O, Saint-Martin C, Gourfinkel-An I, Bouteiller D, Carpentier W, Keren B, Abert B, Gautier A, Baulac S, Arzimanoglou A, Cazeneuve C, Nabbout R, LeGuern E (2009). Spectrum of SCN1A gene mutations associated with Dravet syndrome: analysis of 333 patients. J Med Genet.

[10] Connolly MB (2016). Dravet syndrome: diagnosis and long-term course. Can J Neurol Sci.

[11] Cooper MS, McIntosh A, Crompton DE, McMahon JM, Schneider A, Farrell K, Ganesan V, Gill D, Kivity S, Lerman-Sagie T, McLellan A, Pelekanos J, Ramesh V, Sadleir L, Wirrell E, Scheffer IE (2016). Mortality in Dravet syndrome. Epilepsy Res.

[12] Goncalves C, Martins S, Fernandes L (2021). Dravet syndrome: effects on informal caregivers' mental health and quality of life - A systematic review. Epilepsy Behav.

[13] Strzelczyk A, Kurlemann G, Bast T, Bettendorf U, Kluger G, Mayer T, Neubauer BA, Polster T, von Spiczak S, Trollmann R, Wolff M, Toward T, Gruenert J, Gibson E, Pritchard C, Carroll J, Rosenow F, Schubert-Bast S (2022). Exploring the relationships between composite scores of disease severity, seizure-freedom and quality of life in Dravet syndrome. Neurol Res Pract.

[14] Lagae L, Irwin J, Gibson E, Battersby A (2019). Caregiver impact and health service use in high and low severity Dravet syndrome: a multinational cohort study. Seizure.

[15] Villas N, Meskis MA, Goodliffe S (2017). Dravet syndrome: characteristics, comorbidities, and caregiver concerns. Epilepsy Behav.

[16] Campbell JD, Whittington MD, Kim CH, VanderVeen GR, Knupp KG, Gammaitoni A (2018). Assessing the impact of caring for a child with Dravet syndrome: results of a caregiver survey. Epilepsy Behav.

[17] Jensen MP, Liljenquist KS, Bocell F, Gammaitoni AR, Aron CR, Galer BS, Amtmann D (2017). Life impact of caregiving for severe childhood epilepsy: results of expert panels and caregiver focus groups. Epilepsy Behav.

[18] Jensen MP, Brunklaus A, Dorris L, Zuberi SM, Knupp KG, Galer BS, Gammaitoni AR (2017). The humanistic and economic burden of Dravet syndrome on caregivers and families: implications for future research. Epilepsy Behav.

[19] Nabbout R, Dirani M, Teng T, Bianic F, Martin M, Holland R, Chemaly N, Coque N (2020). Impact of childhood Dravet syndrome on care givers of patients with DS, a major impact on mothers. Epilepsy Behav.

[20] Licheni SH, McMahon JM, Schneider AL, Davey MJ, Scheffer IE (2018). Sleep problems in Dravet syndrome: a modifiable comorbidity. Dev Med Child Neurol.

[21] Van Nuland A, Ivanenko A, Meskis MA, Villas N, Knupp KG, Berg AT (2021). Sleep in Dravet syndrome: a parent-driven survey. Seizure.

[22] von Elm E, Altman DG, Egger M, Pocock SJ, Gotzsche PC, Vandenbroucke JP (2008). The strengthening the reporting of observational studies in epidemiology (STROBE) statement: guidelines for reporting observational studies. J Clin Epidemiol.

[23] Strzelczyk A, Kalski M, Bast T, Wiemer-Kruel A, Bettendorf U, Kay L, Kieslich M, Kluger G, Kurlemann G, Mayer T, Neubauer BA, Polster T, Herting A, von Spiczak S, Trollmann R, Wolff M, Irwin J, Carroll J, Macdonald D, Pritchard C, Klein KM, Rosenow F, Schubert-Bast S (2019). Burden-of-illness and cost-driving factors in Dravet syndrome patients and carers: a prospective, multicenter study from Germany. Eur J Paediatr Neurol.

[24] Buysse DJ, Reynolds CF, Monk TH, Berman SR, Kupfer DJ (1989). The Pittsburgh Sleep Quality Index: a new instrument for psychiatric practice and research. Psychiatry Res.

[25] Doi Y, Minowa M, Uchiyama M, Okawa M (2001). Subjective sleep quality and sleep problems in the general Japanese adult population. Psychiatry Clin Neurosci.

[26] Bjelland I, Dahl AA, Haug TT, Neckelmann D (2002). The validity of the hospital anxiety and depression scale. An updated literature review. J Psychosom Res.

[27] Hinz A, Brahler E (2011). Normative values for the hospital anxiety and depression scale (HADS) in the general German population. J Psychosom Res.

[28] Grau H, Graessel E, Berth H (2014). The subjective burden of informal caregivers of persons with dementia: extended validation of the German language version of the Burden Scale for Family Caregivers (BSFC). Aging Mental Health.

[29] Gräßel E. *Häusliche-Pflege-Skala HPS zur Erfassung der Belastung bei betreuenden oder pflegenden Personen* [Burden Scale for Family Caregivers BSFC for the assessment of subjective burden]. In. Ebersberg: Vless; 2001.

[30] Cohen J (1988). Statistical power analysis for the behavioral sciences.

[31] Hinz A, Glaesmer H, Brahler E, Loffler M, Engel C, Enzenbach C, Hegerl U, Sander C (2017). Sleep quality in the general population: psychometric properties of the Pittsburgh Sleep Quality Index, derived from a German community sample of 9284 people. Sleep Med.

[32] Bailey LD, Schwartz L, Dixon-Salazar T, Meskis MA, Galer BS, Gammaitoni AR, Schad C (2020). Psychosocial impact on siblings of patients with developmental and epileptic encephalopathies. Epilepsy Behav.

[33] Skluzacek JV, Watts KP, Parsy O, Wical B, Camfield P (2011). Dravet syndrome and parent associations: the IDEA League experience with comorbid conditions, mortality, management, adaptation, and grief. Epilepsia.

[34] Strzelczyk A, Schubert-Bast S, Bast T, Bettendorf U, Fiedler B, Hamer HM, Herting A, Kalski M, Kay L, Kieslich M, Klein KM, Kluger G, Kurlemann G, Mayer T, Neubauer BA, Polster T, von Spiczak S, Stephani U, Trollmann R, Wiemer-Kruel A, Wolff M, Irwin J, Carroll J, Pritchard C, Rosenow F (2019). A multicenter, matched case-control analysis comparing burden-of-illness in Dravet syndrome to refractory epilepsy and seizure remission in patients and caregivers in Germany. Epilepsia.

[35] Losito E, Kuchenbuch M, Chemaly N, Laschet J, Chiron C, Kaminska A, Nabbout R (2017). Age-related, "Sleep/nocturnal" tonic and tonic clonic seizure clusters are underdiagnosed in patients with Dravet Syndrome. Epilepsy Behav.

[36] Riemann D, Baum E, Cohrs S (2017). S3-Leitlinie Nicht erholsamer Schlaf/Schlafstörungen. Somnologie.

[37] Backhaus J, Hohagen F, Voderholzer U, Riemann D (2001). Long-term effectiveness of a short-term cognitive-behavioral group treatment for primary insomnia. Eur Arch Psychiatry Clin Neurosci.

[38] Simón MA, Bueno AM, Otero P, Blanco V, Vázquez FL. Caregiver burden and sleep quality in dependent People's family caregivers. J Clin Med 2019;8. 10.3390/jcm8071072 PMC6678125 31336559

[39] Reilly C, Atkinson P, Memon A, Jones C, Dabydeen L, Cross JH, Das KB, Gillberg C, Neville BGR, Scott RC (2018). Child and parental sleep in young children with epilepsy: a population-based case-control study. Epilepsia Open.

[40] Yang H, Feng Y, Zhu Z, Qiao Z, Xiao B, Feng L (2020). Evaluation of anxiety, depression, and sleep quality among parents of children with epilepsy in Southern China. Epilepsy Behav.

[41] Ikeda T, Nagai T, Kato-Nishimura K, Mohri I, Taniike M (2012). Sleep problems in physically disabled children and burden on caregivers. Brain Dev.

[42] Chu J, Richdale AL (2009). Sleep quality and psychological wellbeing in mothers of children with developmental disabilities. Res Dev Disabil.

[43] Li X, Liu M, Lin J, Li B, Zhang X, Zhang S, Lu Z, Zhang J, Zhou J, Ou L (2021). A questionnaire-based study to comprehensively assess the status quo of rare disease patients and care-givers in China. Orphanet J Rare Dis.

[44] Carter PA (2006). A brief behavioral sleep intervention for family caregivers of persons with cancer. Cancer Nurs.

[45] Strzelczyk A, Schubert-Bast S (2022). A practical guide to the treatment of Dravet syndrome with anti-seizure medication. CNS Drugs.

[46] Lagae L (2021). Dravet syndrome. Curr Opin Neurol.

[47] Strzelczyk A, Schubert-Bast S (2022). Psychobehavioural and cognitive adverse events of anti-seizure medications for the treatment of developmental and epileptic encephalopathies. CNS Drugs.

